# Observations of the development of *Xanthoparmelia farinosa* under optical and electron microscopy

**DOI:** 10.1080/21501203.2017.1367333

**Published:** 2017-08-22

**Authors:** Renato Andrés García, Vilma Gabriela Rosato

**Affiliations:** a Laboratorio de Entrenamiento Multidisciplinario para la Investigación tecnológica, La Plata, Argentina; b Consejo Nacional de Investigaciones Científicas y Técnicas, Argentina; c Universidad Tecnológica Nacional, Facultad Regional La Plata, La Plata, Argentina

**Keywords:** Lichen, outdoor, soralia, glass, adhesion to the substrate, lobe formation

## Abstract

*Xanthoparmelia farinosa* is a foliose lichen widely distributed in South America, growing not only on rocks but also on man-made structures. This species has abundant soralia, but it is unknown how development occurs from the soredium to the formation of a complete thallus. The soredia were extracted from the thallus with forceps, planted on glass plates and exposed to outdoor conditions for a period of 24 months; in every 3 months, optical inspection was performed with a stereomicroscope and a compound microscope, in addition, four samples with different exposure times were chosen to observe under a scanning electron microscope. The development of hyphae and the adhesion of these to the substrate, and the outlines of the formation of the lobules and rhizines could be observed. Our study is a first attempt to understand the development of this species which is endemic to South America and very common in the area.

## Introduction

Foliose lichens have a flat continuous thallus and radial growth, with a distinctive upper cortex, and a lower cortex attached to the substrate, usually by rhizines. Many foliose thallus margins consist of individual marginal lobes exhibiting radial growth and periodically divided; this division can give lobules of equal or different size and the result is a complex structure (Aplin and Hill 1979; Hooker 1980; Hill 1984; Armstrong 1991a, 1993). In foliose lichens, the forms of vegetative reproduction are isidia, soredia and thallus fragments (Armstrong 1981, 1990, 1991b). Within these, the soredia consist of a few algal cells enveloped by a loose mantle of hyphae; these are small and with a range of 50 mm in diameter. They can be in areas delimited on the upper surface named soralia or scattered without grouping. The soredial masses are released from the soralia and dispersed by either physical disruption or by hygroscopic movements of cortical tissue (Jahns et al. 1976; Büdel and Scheidegger 2008). The soredia are often hydrophobic; the repulsion by water droplets may result in their dispersal from soralia. The size and shape of the resulting thallus depends on the number of soredia involved in its formation, and this is frequently scattered in groups of various sizes (Armstrong 1990).

Honegger (2008) proposes that the vegetative propagules the first structure that this form is a prethallus, a crustose structure without internal differentiation. At this stage, the formation of the thallus begins, characterised by the formation of layers, growth areas and areas of no growth, algal layer formation and formation of secondary metabolites, among other events. But she also recognises that the stimulus to pass from prethallus state to the formation of thallus is so far unknown. Stocker-Wörgötter (1991) studied the development of *Peltigera didactyla* and *Peltigera praetextata* from soralia; he used sterilised soil from the habitat of the lichen as substratum and laboratory conditions simulating environmental conditions. He observed that the germination of the soredia took place during a time interval of 2–4 weeks, and the mature thalli were observed between 5 and 6 months.

Hilmo and Ott (2002) cultivated soredia of *Lobaria scrobiculata* and *Platismatia* sp. on clean branches and outdoor conditions; they observed a slow development of thalli from vegetative diaspora, at least 4 years was required for the development of a recognisable juvenile thallus. In *L. scrobiculata*, for example, the first distinguishable lobe was observed 29 months after the start of the experiment that lasted 4 years. Authors suggest that low temperatures and snow covering the diaspora might explain the long period for juvenile development, which also explains the slow colonisation.

Scheidegger (1995) used cotton gauze disc as artificial substrate for propagules of *Lobaria pulmonaria*, these were exposed to environmental conditions and periodically removed for observation. He found that in *L. pulmonaria*, anchor hyphae developed 2–4 months after germination. After 15 months, the growth areas were differentiated and lobules 0.5 mm wide appeared. Zoller et al. (2000) used a similar culture method for isidia and soredia of three species of corticulous lichens; these were exposed to environmental conditions and periodically removed for observation. First lobes resembling adult thalli were observed after 8–12 months in *Sticta fuliginosa* and *Leptogium saturninum* but only after 16 months in *Menegazzia terebrata*. In both studies, the authors not only provide basic information on the development of lichens but also propose this method to reinsert species whose populations are in danger.

Schuster et al. (1985) cultivated soredia under outdoor conditions on bark; in all cases, they observed that an extended network of hyphae secured soredia to the substrate as well as joined other soredia to form a gelatinous tissue. They observed the formation of lobes on *Parmelia sulcata, Usnea filipendula* and *Hypogymnia physodes* after 12 months, whereas in *Physcia tenella*, they were seen at 9 months and rhizines were developed before the formation of lobes.

Anstett et al. (2014) managed to grow two species of the lichen *P. sulcata* and *Physcia ascendens* on plastic cover slips and outdoor conditions in order to observe the development of soredia. They noted that aposymbiotic hyphae that adhered the mass of soredia at the substrate appeared after 2 weeks, under favourable conditions. After 6 months, they observed pigmentation, rhizines and development of epicortex. They also observed that cold temperatures do not necessarily suspend lichen development.

The aim of this work is the description of the early stages of development of *Xanthoparmelia farinosa* from soredia.

## Materials and methods

**Figure 1.**
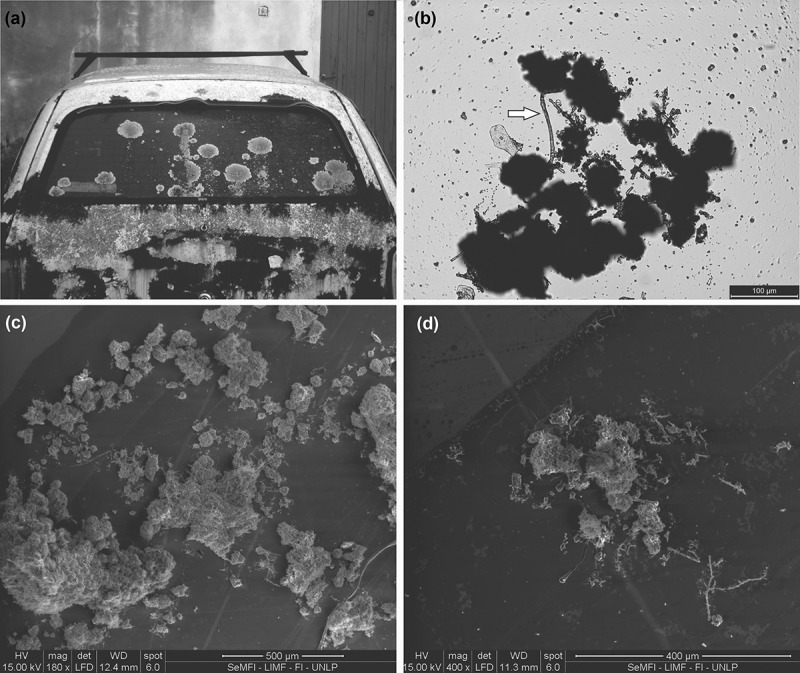
(a) *X. farinosa* growing on car glass with 15 years of abandonment. (b) *CM silhouette image*. White arrow marks the union of the masses with a hypha. (c) *SEM image*. Soredia grouped in different size. (d) *SEM image*. Hyphae projecting from the mass of soredia.

The species selected for this study was *X. farinosa*, this is a South American saxicolous lichen, with a foliose yellowish green tallus, the upper surface is smooth and emaculate; with soralia punctiform, coalescent and sparse over the lamina; lower surface with sparsely rhizines (Nash et al. 1995). This species can grow on rocks and building materials such as ornamental rocks, mortar and glass (Figure 1(a)) (Guiamet et al. 2012; García et al. 2014).

Using a stereoscopic microscope and forceps, soredia were extracted from soralia and seeded on glass slides 7.5 × 2.5 cm. A drop of distilled water to the mass of soredia was added and then disaggregated into small groups with tweezers. Before exposure, they were allowed to stand 2 days in indoor conditions so that the water evaporates, helping soredia adhere to the surface (Ansett et al. 2014).

The glass plates were attached to a dark plastic plate, which prevented the light from passing, so that the soredia only received light from one side, avoiding possible deformations (Vogel 1955). The plates were directed towards the ground an inclination of 45°, facing NW, to avoid dragging the soredia by rain and wind. The plates were placed on the roof of Laboratorio de Entrenamiento Multidisciplinario para la Investigación Tecnológica in the La Plata city (34°56′00″S 57°57′00″W), Buenos Aires province, Argentina. The city has a humid temperate climate with rainfall throughout the year. The average annual temperature is 16.3°C and precipitation is 946 mm per year (www.climatedata.org).

The plates were observed at 2 days (before placing them outside), at the first month and every 3 months for a total of 24 months, to generate the least possible stress, collecting of plates and observations conducted on the same day. Observations were made under compound microscope (CM) and stereomicroscope (SM). The observations were made with the SM from above; the glass plate was also inverted and observed from below. Under CM, the silhouette of soredia and hyphae was observed. All the observations were made with the dry material, to preserve the adhered soredia; once the observations were made, the glass plates were exposed again to environmental conditions. The observation was done in a single day to cause the least possible stress.

Observations were also performed under a scanning electron microscope FEI, Quanta 200. We opted for low vacuum technique, because it allows the chosen samples to be left again in outdoor conditions to continue studying their growth.

## Results

Of the 10 plates seeded at the beginning of experiment, only 7 remained which only contained 3 or 4 masses of soredia of the initial approximately 20 soredia groups each. So, this caused a loss of 90% of soredia. There was no evidence that the loss has been caused by animal feeding, so we interpreted that the loss of this mass of soredia is caused by the lack of an efficient attachment structure.

Concerning the development of structures from the soredia, the following events are observed:

After 2 days, the soredia did not present morphological changes; under microscope, it was possible to observe that all the groups of soredia presented first short and hyaline hyphae. And the union of groups of soredia by hyphae was recorded, although rare (Figure 1).

After a month, it was observed that the hyphae had multiplied all along the edge of the mass of soredia; some hyphae of greater extension were formed and some unions between the masses of soredia were observed (Figure 1).

**Figure 2.**
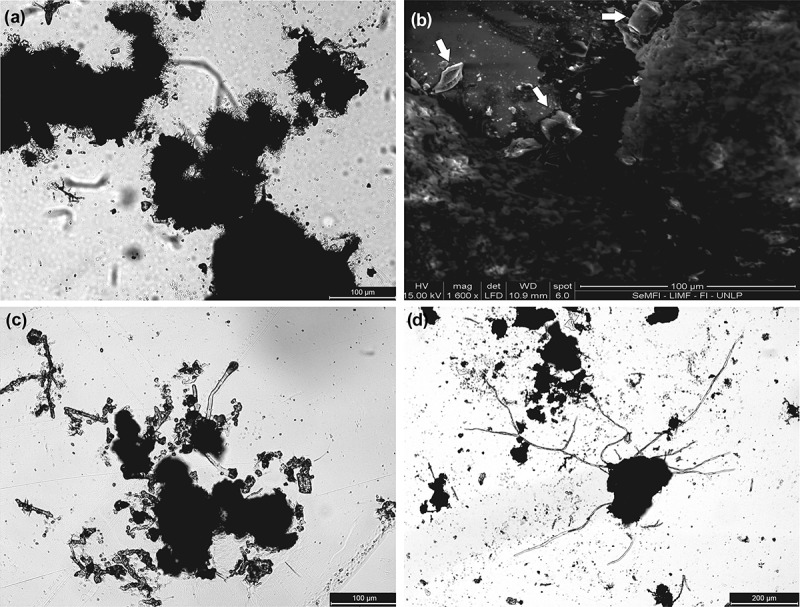
(a) *CM silhouette image*. Hyaline and short hyphae covering the edges of the mass of soredia. (b) *SEM image*. Sand crystals adhered to the substance exuded by the mass of soredia. (c) *CM silhouette image*. Long hyaline hyphae in early stages. (d) *CM silhouette image*. Long hyaline hyphae in branching.

After 3 months, it was observed that hyaline hyphae of 30 μm were spreading from the edge of the mass of groups. Due to their position, time of onset and high density of hyphae, we believe that would be part of the structure of primary anchor. Also on the hyphae, particles of sediment were observed to stick on, so these hyphae would release an adhesive substance. At this point, no more soredia were observed, since all those that were found were united in a single mass (Figure 2).

After 6 months, the hyphae are anastomosed between them forming networks and linking masses of soredia. They were primarily hyaline hyphae as well as other long hyphae, thickening and darkening from the base. The soredia forming groups begin to lose individuality, and the beginning of fusion can be seen (Figure 2).

**Figure 3.**
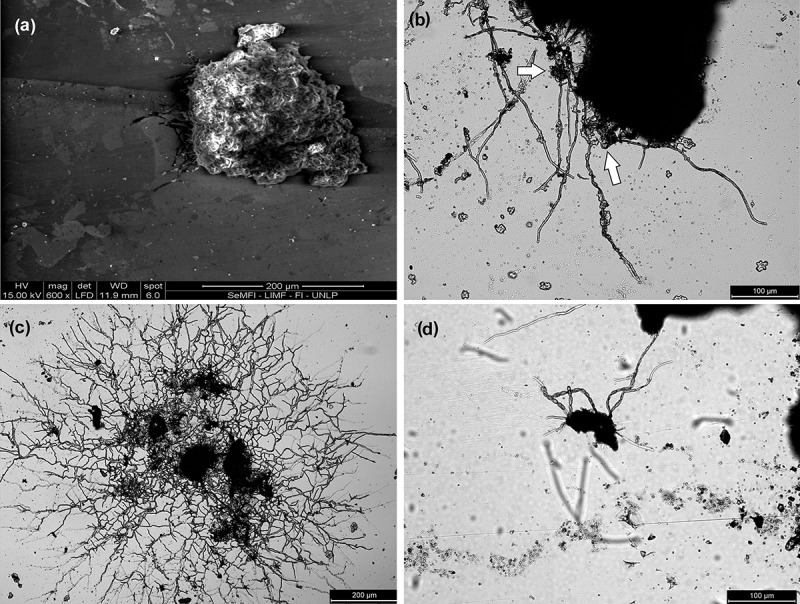
(a) *SEM image*. Short anastomosing hyphae. (b) *CM silhouette image*. White arrows mark long hyphae trapping algae chlorophytes. (c) *CM silhouette image*. Arachnoid state, branched and anastomosed hyphae. (d) *CM silhouette image*. Hyphae in the process of thickening.

After 9 months, the beginning of thickening seen in the previous observation was extended, covering the hyphae and extending to other hyphae. A lot of dark thickened hyphae of up to 0.5 mm long were observed. Thickened hyphae exuded an unidentified substance; hyphae were observed to meet *Chlorophyta* algae that were growing on the plate, contact and capture them. Also, the amount of binding hyphae, which spread between soredia groups, is increased (Figure 3).

**Figure 4.**
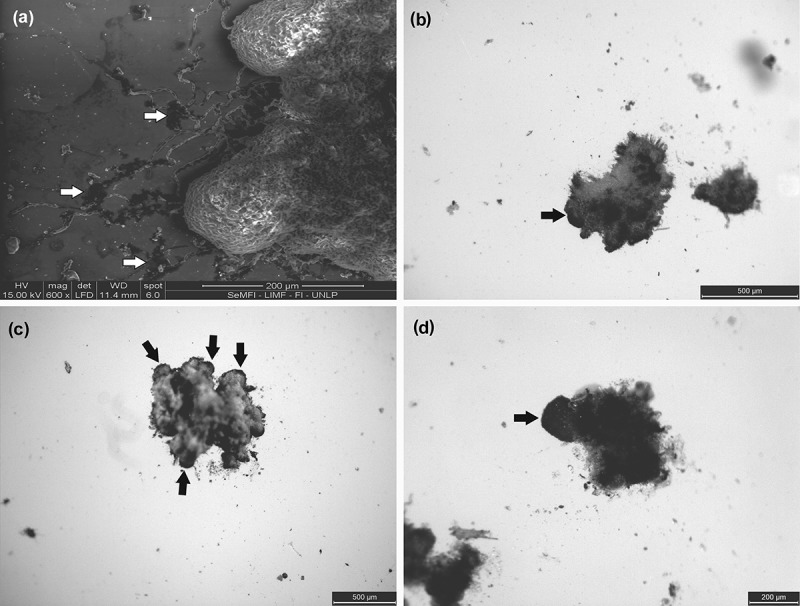
(a) *SEM image*. White arrows mark the lysis of hyphae coming out of the protolobules. (b) *SM image from below*. The black arrow marks the protolobule. (c) *SM image from above*. The black arrows mark the protolobules. (d) *SM image from below*. The black arrow marks the lobule.

After 12 months, increased fusion between soredia was observed, almost no soredia can be individualised. The edges of this mass of soredia darkened and began to take defined lobed form (protolobe); hyphae are lost in these sectors by cell lysis, and the upper cortex begins to form, which is still not a continuous layer. In the contact zone between the mass of soredia and the substrate, a discontinuous layer of dark colour was formed, considered to be the outline of the lower cortex. We believe that this step is the formation of protolobe (Figure 4).

After 18 months, it was observed in some sectors that the edge became circular and it acquires a dark pigmentation; also, the upper part becomes smoother differentiating of the mass of soredia that gave rise to it (protolobes), although not all groups developed soredia this stage. At this stage, the appearance of the first lobe is observed, with a formed lower and upper cortex, being greater in size 0.28 × 0.21 observed mm. Once the lower cortex is formed, begin the formation of rhizines. In addition, from the rhizines, hyaline hyphae were seen in the region of contact with the substrate (Figure 4).

**Figure 5.**
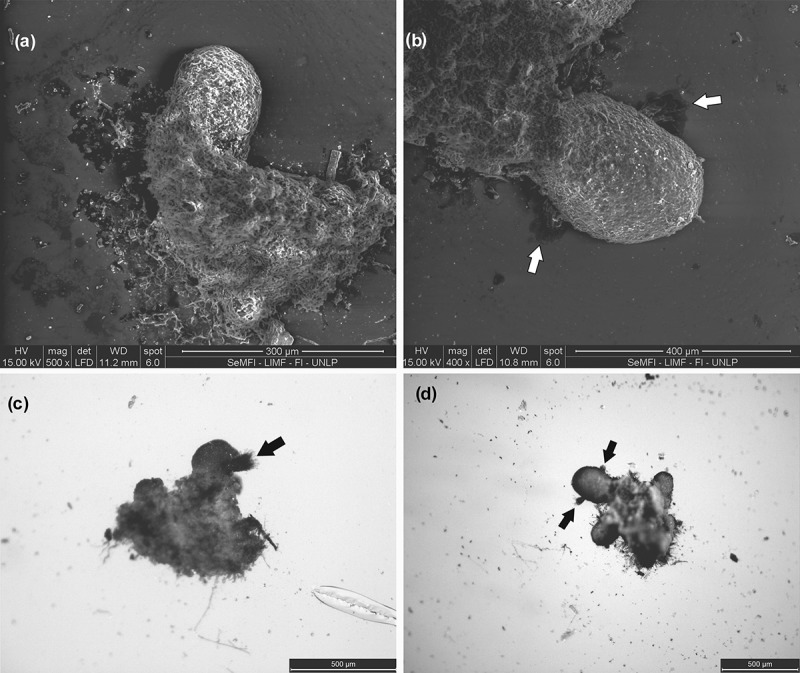
(a) *SEM*. formed lobule. (b) *SEM*. White dates mark the rhizines. (c) *SM from below*. Black arrows mark the rhizin. (d) *SM from above*. Black arrows mark rhizines.

After 24 months, no major changes were observed, the protolobules formed lobules and new sectors of protolobules were observed (Figure 5).

## Discussion

The full grow thallus of *X. farinosa* has numerous soralia with soredia. In this experiment, only 10% of all masses of soredia have been able to remain attached, even with the conditions of protection given to encourage their anchorage. Zoller et al. (2000) were able to obtain a high soredia establishment on a rougher surface as gauze discs. Although it is possible that the high loss of soredia is due to the smooth surface; the utility of the method for observing the development of this species in a simple way was confirmed. The most successful fixation has been the masses of sorendia and not the solitary soredium, possibly this type of smooth surface favouring to the masses of soredia since they will have more surface of grip than the solitary ones. Maybe it would be necessary to abrade the surface of the glass in a future experiment, to create a rough surface that allows a better adhesion. But on the other hand, Stocker-Wörgötter (1991) suggests that a single soredia often degenerated within several weeks after germination and groups or several agglomerations of soredia were necessary for thallus formation. So, it is possible that the substrate is not the problem, but the subsistence depends on the number of soredia that form the groups. What remains without knowing is how many soredia are necessary for the formation of a thallus.

As it was observed by Schuster et al. (1985), Scheidegger (1995) and Anstett et al. (2014), the first visible structure is the hyaline hyphae with anchor function; Schuster et al. (1985) termed this stage “arachnoidal stage” characterised by radiating hyphae. In our case study, we consider that this arachnoid stage appears around 6 months, which Anstett et al. (2014) termed aposymbiotic hyphal extension. This is the most important stage because if they do not adhere properly, soredia can be swept away by rain or wind.

For *X. farinosa*, these anchoring hyphae are hyaline and short. We do not consider these hyphae to be aposymbiotic. Although they are not directly associated with algae, the hyphae arise from a symbiotic mass. We do not consider that these hyphae are a aposymbiotic: although the algae would not be participating in this structure, we believe that their origin is a structure of the symbiosis (soredia) and these hyphae are part of it, so we prefer to call them hyaline hyphae although we understand that they would be similar to those observed by Anstett et al. (2014).

Like Hilmo and Ott (2002), we found a gelatinous substance exuded from the hyphae, but different from Honegger (1987) and Kon and Ohmura (2010) who observed the gelatinous substance from the cortex of the soredia. We agree with these authors that the function of this gelatinous substance is the substrate adhesion; it appears in the first few months to help with adhesion and we found that it also caught sand grains.

Subsequently, we observed that some of the hyaline hyphae changed to a thick, dark shape. It was also noted that in some cases, these trapped algae joined masses of soredia and exuded an adhesive substance. Whereas the existence of soredia groups joined by hyphae and hyphae trapping algae species has been reported (Schuster et al. 1985; Honegger 2008), no mention has been made of a morphological difference between the exclusive anchoring hyphae and binding hyphae as we have seen here. So, we consider this morphological type as exploration hyphae.

We also observed the formation of a stage preceding the formation of the lobule, which has been nominated by Ansett et al. (2014) as protolobe. In *X. farinosa*, this state was defined by having a proper edge, an upper cortex formation with traces of pre-existing soredia and a lower cortex formation presents the dark colour characteristic of the species but has some spaces. The hyphae that extended from this part were lost. The hyphae suffered a cellular lysis, since the structures of fixation are replaced by the fixation of the inferior cortex in formation and later by the rhizines.

As regards their development, the lobes of *X. farinosa* grow slower compared to other species of foliose lichens as *P. tenella, H. physodes, P. sulcata, L. pulmonaria, P. didactyla* and *P. praetextata* (Schuster et al. 1985; Stocker-Wörgötter 1991; Scheidegger 1995; Anstett et al. 2014), although the development was faster than that observed for *L. scrobiculata* (Hilmo and Ott 2002). Contrary to that observed by Schuster et al. (1985) for *P. tenella*, rhizines formation in *X. farinosa* occurs after the formation of lobes and lower cortex. As the studies have been carried out on different species and in different types of climate, it is expected that the same ones do not have a similar speed of development.

Many techniques have been used to be able to study the development of soredia; among these, most of the authors have preferred to leave the cultures under environmental conditions (Schuster et al. 1985; Scheidegger 1995; Hilmo and Ott 2002; Anstett et al. 2014), except by Stocker-Wörgötter (1991) who has chosen to use controlled light and humidity in the laboratory. Both types of experiments have shown favourable results, although the use of outdoor conditions leads to greater simplicity since it takes several months to observe results. On the other hand, other authors have chosen to use the substrate that is preferred by the species (Schuster et al. 1985; Stocker-Wörgötter 1991; Scheidegger 1995; Hilmo and Ott 2002), but this methodology complicates the moment of observations with fast and simple methods like the microscopes, because for each observation, material must be removed and it is lost. Cultivation on a transparent material such as glass has shown a clear advantage by allowing to observe the development of *X. farinosa* with traditional optical methods like the microscope and to observe the development of the lower cortex that would have been impossible to observe on another type of substrate. However, since the artificial substrates cannot be favourable for all species, it is necessary to know if the species to be studied grows and develops correctly in this type of substrate.

It is noteworthy that this work is a first attempt to understand the development of a complete thallus from soredia. It is planned to continue to track these thalli to see their continuous development to fertile thallus. It would be interesting to know if it is possible to use this procedure with other species, so maybe in the future to perform transplants with more developed thallus.

## Conclusion

Although similarities have been found between the stages of development of the different foliose lichens, large differences have also been found. So, we believe that generalisations can not be made, but each species presents its unique pattern. As conclusion, we can differentiate six stages of development of *X. farinosa*.

1. *Attachmentstage*. This is the first step, after a few days small hyaline attachment hyphae appear, which keep the mass of soredia attached to the surface.
2. *Arachnoid stage*. Occurs in the first months, hyphae are prolonged by exploring in a circular way the mass of soredia. The hyphae are joined together forming a net and joining the masses of sorceries.
3. *Thickening hyphae stage*. This stage occurs from 6 months until the destruction of the hyphae. The long hyphae suffer a thickening process and acquire a brown colour from the base to the apex that remains in division.
4. *Protolobe stage*. At this stage, the hyphae are lost, the edge takes on the characteristic circular shape. It begins to form the upper cortex with a texture less rough and more even than that of the mass of soredia, the lower part begins to darken giving origin to the future lower cortex.
5. *Lobule stage*. This stage is characterised by the formation of the lobule; it is an extension of circular form differentiated from the mass of soredia that gives rise to it, is raised on the substrate, its upper and lower cortex has a homogeneous and smooth appearance, and the lower cortex presents the characteristic colour of the species.
6. *Rhizine stage*. At this stage, the lobes are already formed and from the lower cortex origin, the rhizines will attach the thallus to the substrate.
