# Corrigendum

**DOI:** 10.1080/21501203.2017.1380353

**Published:** 2017-10-16

García RA, Rosato VG. 2017. Observations of the development of *Xanthoparmelia farinosa* under optical and electron microscopy. Mycology. https://doi.org/10.1080/21501203.2017.1367333

When the above article was first published online, the species name *Xanthoparmelia farinosa* was set incorrectly as *Xanthoria farinosa* in the title and abstract. This has now been corrected in both the print and online versions.

The authors apologize for this error.

